# Clinical Radiographical Outcomes and Complications after a Brand-New Total Ankle Replacement Design through an Anterior Approach: A Retrospective at a Short-Term Follow Up

**DOI:** 10.3390/jcm10112258

**Published:** 2021-05-23

**Authors:** Massimiliano Mosca, Silvio Caravelli, Emanuele Vocale, Simone Massimi, Davide Censoni, Marco Di Ponte, Mario Fuiano, Stefano Zaffagnini

**Affiliations:** II Clinic of Orthopaedic and Traumatology, Istituto Ortopedico Rizzoli, Via Dei Colli, 10-40136 Bologna, Italy; massimiliano.mosca@ior.it (M.M.); emanuelevocale@gmail.com (E.V.); dottmassimi@gmail.com (S.M.); censonidavide@hotmail.it (D.C.); marco.diponte@gmail.com (M.D.P.); mariofuiano@yahoo.it (M.F.); stefano.zaffagnini@unibo.it (S.Z.)

**Keywords:** total ankle replacement, new design prosthesis, end-stage osteoarthritis, anterior approach

## Abstract

Recently, the progress in techniques and in projecting new prosthetic designs has allowed increasing indications for total ankle replacement (TAR) as treatment for ankle osteoarthritis. This retrospective work comprehended 39 subjects aged between 47 and 79 years old. The patients, observed for at least 12 months (mean follow up of 18.2 ± 4.1 months), have been evaluated according to clinical and radiological parameters, both pre- and post-operatively. The AOFAS and VAS score significantly improved, respectively, from 46.2 ± 4.8 to 93.9 ± 4.1 and from 7.1 ± 1.1 to 0.7 ± 0.5 (*p* value < 0.05). At the final evaluation, the mean plantarflexion passed from 12.2° ± 2.3° to 18.1° ± 2.4° (*p* value < 0.05) and dorsiflexion from a pre-operative mean value of 8.7° ± 4.1° to 21.7° ± 5.4° post-operatively (*p* value < 0.05). This study found that this new total ankle replacement design is a safe and effective procedure for patients effected by end-stage ankle osteoarthritis. Improvements have been demonstrated in terms of range of motion, radiographic parameters and patient-reported outcomes. However, further studies are needed to assess the long-term performance of these prostheses.

## 1. Introduction

Ankle osteoarthritis (AO) constitutes a large burden to society and it is a leading cause of chronic disability in an increasing part of the world’s population [1]. This pathological entity presents nonspecific symptoms of stiffness, swelling, and pain. Most commonly, it is secondary to traumas, it occurs in younger individuals, and it is associated with obesity, metabolic disease, chronic inflammatory joint diseases, septic arthritis and anatomical pathological variations that can be responsible for biomechanical disfunctions.

The first treatment line for AO is represented by non-operative options, such as weight loss, physical therapy, bracing, orthoses, pharmacological treatments, corticosteroid injections, and visco-supplementation; however, if these measures prove to be ineffective, a surgical treatment must be considered [2].

Thanks to advances in the use of materials and techniques, new prosthetic designs are always being designed for the market. Advances have been made also to improve implantation techniques and provide more sophisticated surgical instrumentation.

Recent implants for total ankle replacement (TAR) are classified into the following two categories: two-component prostheses with fixed meniscus and three-component prostheses with mobile meniscus [3]. In fix-bearing prostheses, where the meniscal component is fixed to the tibial one, the rationale consists in the greater capacity of this implant to absorb rotational forces. On the other hand, the mobile-bearing prosthesis is characterized by a mobile meniscus that articulates with both the talar and tibial components, reducing shear forces and presenting better adaptability to micromovements. Mobile-bearing prostheses combine congruency with weakly bound components to let the soft tissues guide the physiological motion of the tibiotarsal joint [4].

New generation prostheses are characterized by a talar resurfacing component, a tibial component (implantable with minimal tibial resection) and a mobile meniscus, which allow the restoration of physiological rotation centers and the ligamentous balance.

The purpose of this work is to assess the effectiveness and the efficiency of total ankle replacement (TAR) with the brand-new design Exatech Vantage^®^ (Exactech, Gainesville, FL, USA), expressed as clinical, radiological and functional parameters, and possible post-operative complications at an early term follow-up. This innovative design of a prosthesis has been implanted through an anterior approach, in patients affected by severe degenerative ankle arthropathy. 

## 2. Materials and Methods

This is a retrospective observational study. Informed consent was obtained from all patients before the surgery was scheduled, following the Declaration of Helsinki. All the data were treated with maximum confidentiality. 

A retrospective data revision from the IRCCS Istituto Ortopedico Rizzoli database was carried out in order to find subjects effected by severe ankle osteoarthritis and treated with the Exatech Vantage^®^ (Exatech, Gainesville, FL, USA) TAR through anterior approach from February 2019 to December 2019. Patients suitable to undergo the surgical treatment included subjects affected by primary ankle osteoarthritis, secondary post-traumatic degenerative arthropathy, and joint degenerative sequelae of rheumatoid arthritis (RA). 

Clinical outcomes considered for this study were the American Orthopedic Foot and Ankle Society (AOFAS) ankle-hindfoot score [5] and the visual analogue scale (VAS) for pain. The joint range of motion (ROM) of the ankle was expressed in terms of degrees of maximum dorsiflexion and plantarflexion (this last evaluation has been conducted using a goniometer).

Weightbearing X-rays, including antero-posterior, mortise and lateral projections of the ankle, were pre-operatively executed to check the alignment of the tibiotarsal joint and hindfoot. Every X-ray evaluation has been conducted using the institutional PACS (picture archiving and communication system—Carestream Health, Rocester, NY, USA). Radiographic parameters considered pre-treatment were as follows: tibiotalar surface angle (normal value 87.2° ± 2.8°), to assess intra-articular misalignments, and Saltzman or HAVA (normal angle 0–5°) to evaluate possible extra-articular hindfoot deformities.

Complications have been classified according to Glazebrook et al. as low-grade (intra-operative bone fractures, wound healing problems), medium-grade (technical errors, bone subsidence, postoperative bone fractures) and high-grade complications (deep infections, aseptic loosening, implant failures) [6].

Exclusion criteria for this work were represented by presence of current peripheral neuropathies, coronal plane deformities greater than 15 degrees, bilateral ankle arthritis, contralateral total ankle replacement, avascular necrosis of the distal tibia or talus and severe talar dome deformities (“flat-talus”). 

Additional procedures are necessary when severe soft tissue deformities (for example the Achilles tendon retraction) or articular/extra articular deformities (such as hindfoot or midfoot deformities, subtalar or midfoot osteoarthritis) are associated with A.O. 

All surgical procedures were performed by the same highly experienced senior foot and ankle surgeon (M.M.)

### 2.1. Surgical Technique

The patient is positioned supine, usually using a small bump under the ipsilateral hip to reduce the external rotation of the interested limb. Cutaneous incision is performed 1 cm lateral to the tibial crest, starting 6–8 cm proximal and ending 6 cm distal to the articular line of ankle joint. After dissecting the subcutaneous tissues, the superficial peroneal nerve is identified at its distal course. The small medial branches of this nerve can be sacrificed, without cutting the whole peroneal nerve. 

Then the upper extensor retinaculum is identified and the extensor hallucis longus (EHL) sleeve is opened. The anterior tibial tendon (ATT) sheath is saved. Once the EHL tendon sheath is open, the anterior neurovascular bundle is positioned below the tendon itself. This gentle anatomical structure is softly moved laterally with EHL tendon and muscle. Successively, the capsule of the ankle joint is widely opened in order to show the entire joint, from the medial to the lateral malleolus. Osteophytes can be removed from the tibial and the talar borders of the joint using an osteotome, enhancing the exposure. 

The ankle’s alignment is determined positioning the “alignment guide”, with attached the tibial cutting block. The bone cuts on tibial and talar side, the prosthesis’ preparation and its positioning are performed assisted by an image intensifier. In order to avoid the joint’s overstuff, great care must be given to the bone resection trying to obtain an appropriate tibial slope and a correct talar positioning on the sagittal plane. A curved rasp is used to refine irregular spots on the talus to get a regular rounded surface that will suit to the talar component.

**Figure 1:**
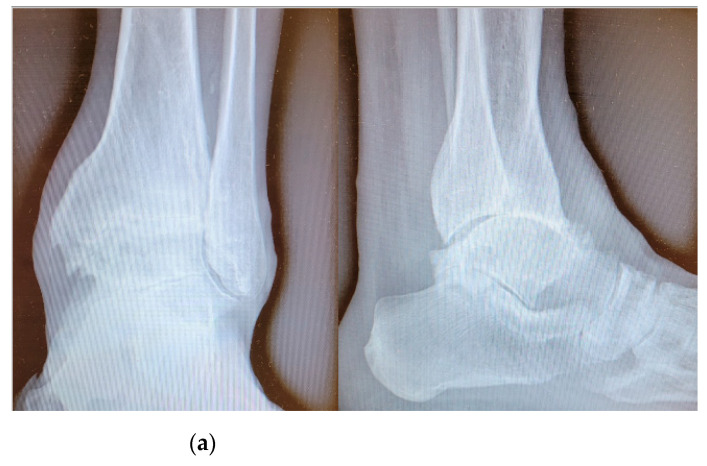

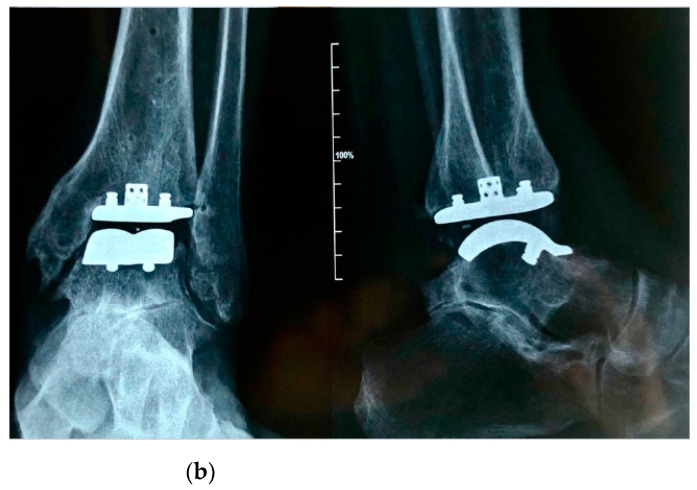
Pre-operative radiographic image in anteroposterior and lateral view (on the left and on the right, respectively) of an arthritic ankle joint, with a slight articular varus deformity (**a**) and post-operative radiographic results (**b**).

**Figure 2:**
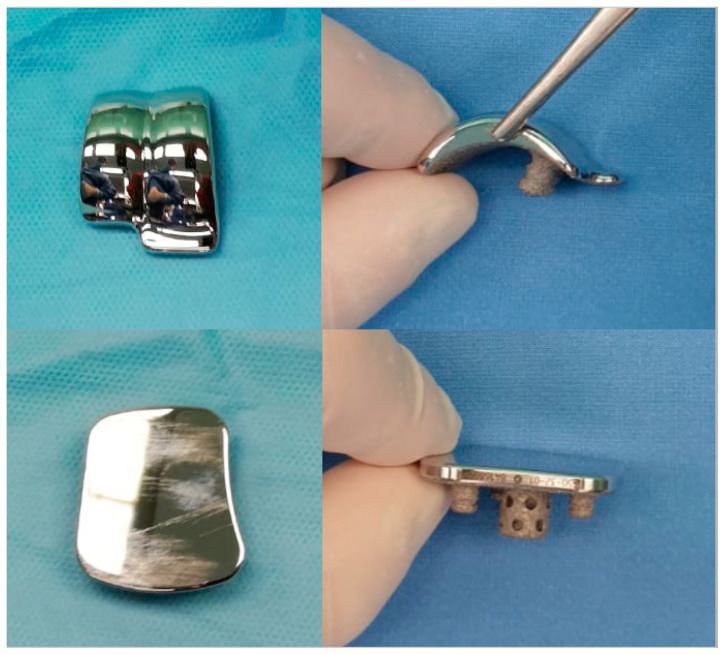
Prosthetic components. It is to be noted, the new design *silhouette* and the fixation system of the talar and tibial components.

After preparing the talar and the tibial surfaces, the trial can be fixed with a screw and the alignment is fluoroscopically checked. Trial components point out the correct position of the prosthesis, the appropriate size of the implant, the stability and the ROM. Once the position and the size of the prosthetic components are defined, the final components can be implanted with a press-fit fixation (Figure 1 and Figure 2). 

### 2.2. Accessory Procedures

- *Achilles tendon lengthening*: to be performed in case of retraction of the Achilles tendon.
- *In-varus calcaneal osteotomy*: to execute if valgus deformities of the hindfoot are present.
- *Hindfoot and midfoot arthrodesis*: fusion of subtalar joints, midtarsal joints and triple arthrodesis, if arthrosis of the foot articulation is present.

### 2.3. Post-Operative Treatment 

After the surgical treatment, if accessory procedures were not performed, a Walker boot is positioned for four weeks with partial weight-bearing during the walk on the operated limb. The brace can be removed more times per day, in order to exercise dorsi-flexion and plantar-flexion, both actively and passively. After 21 days post-surgical treatment, a progressive increase in the weight-bearing to full weight placed on the operated ankle is permitted. Generally, patients can walk without auxiliary aids in a month after the surgical intervention. If accessory bone procedures have been performed, a cast is positioned for two weeks with no weight-bearing walking on the affected limb. At two weeks post-operative, the orthopedic brace may be removed several times a day to perform active and passive mobilization exercises on the ankle. At four weeks post-operative, a partial weight-bearing on the operated limb is allowed. 

### 2.4. Statistical Analysis

The variables employed to characterize the sample were continuous. They have been reported as mean and standard deviation (SD). 

Repeated-measures study of variance was performed to assess tendencies in clinical outcomes at follow-up. A single-way evaluation of variance was executed to analyze the difference among scores if the variables adhered to a Gaussian distribution, using the T-Student test. Differently, the Wilcoxon rank test was used. 

Shapiro–Wilk test has been applied to verify if the data analyzed were characterized by a Gaussian distribution; *p* value < 0.05 was considered as significant.

Statistical analysis was performed using the statistical package for the social sciences (SPSS) software version 25.0 (IBM Corp., released 2017. IBM SPSS Statistics for Windows, version 25.0. Armonk, NY, USA, IBM Corp). 

## 3. Results

The data of 40 consecutive patients effected by high-grade ankle osteoarthritis and treated with primary TAR through the anterior approach (Exactech Vantage^®^ Total Ankle System), from February 2019 to December 2019, were analyzed. Each surgical procedure was executed by an experienced foot and ankle surgeon (M.M.). One patient did not give his consent to the study. Thirty-nine patients (23 females, 16 males) aged between 47 and 79 years old (mean age 57.2 ± 5.9) were included for this study. Fourteen (35.8%) patients underwent associated procedures (two calcaneal osteotomies for valgus hindfoot, one calcaneal osteotomy for varus hindfoot, one fibular lengthening procedure for valgus ankle, two subtalar arthrodeses and eight Achilles tendon lengthenings). 

The average follow-up was 18.2 ± 4.11 months, varying from a minimum of 12 months to a maximum of 22 months. 

The AOFAS score significantly improved from 46.2 ± 4.8 (48–72) pre-treatment to an average post-operative score of 93.9 ± 4.1 (67–97) (*p* value < 0.05). The VAS scale improved in pain from a pre-treatment mean value of 7.1 ± 1.1 (9–6) to a post-operative average value of 0.7 ± 0.5 (2–0) (*p* value < 0.05). 

**Figure 3:**
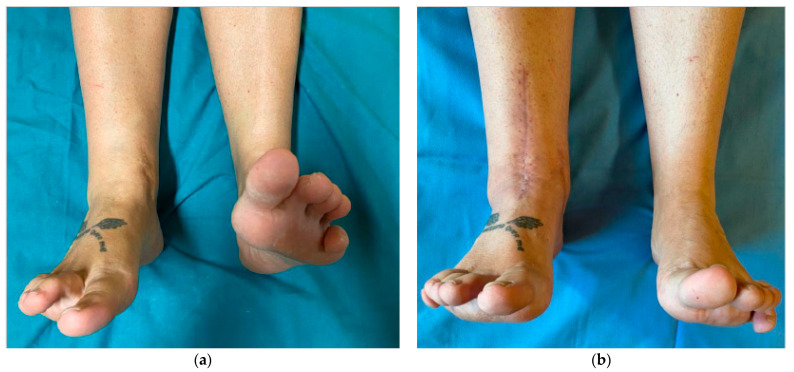
Pre-operative clinical picture, with an evident range of motion limitation (**a**) and post-operative clinical results in terms of movement restoration (**b**).

At the final follow-up, the mean plantarflexion passed from 12.2° ± 2.3° to 18.1° ± 2.4° (*p* value < 0.05), and dorsiflexion from a pre-operative mean value of 8.7° ± 4.1° to a mean value of 21.7° ± 5.4° post-operatively (*p* value < 0.05) (Figure 3). 

Five post-operative wound healing delays (low-grade complications according to the Glazebrook classification) [6] have been registered as minor complications in our case series (12.8%). Two of them healed through simple dressing; the other three cases were characterized by superficial infections, and they were treated with broad-spectrum antibiotic therapy and advanced dressings. At the final follow-up, all the X-ray’s controls showed no radiographic signs of early loosening, dislocation of the insert, nor alignment changes compared to the post-operative X-ray check. No medium-grade complications (technical errors, bone subsidences) and no high-grade complications (deep infections, aseptic loosening) have been described. There were no complications due to the associated procedures performed on the bones or on the soft tissues.

## 4. Discussion

AO is a disabling pathology that can cause severe pain, discomfort and limitation of daily activities. The TAR is a relatively young surgery, its results have not been always satisfying in the past. This represented the silver lining for more traditional interventions, such as ankle arthrodesis [7].

The advent of TAR has made it possible to give partial recovery of motility to the patient, allowing a more biomechanically correct gait in order to preserve the adjacent joints over time [8]. The scientific literature shows that TAR represents a viable alternative to arthrodesis in reducing pain, improving joint motility, improving daily life, and reducing the incidence of complications [9].

The design of the ankle prostheses has been updated and developed since the first models in the 1970s. The prosthesis used by the authors for this study represents a highly innovative design aimed to restore, as much as possible, a physiological anatomy of the tibiotalar joint. The rationale of this design is based on the assumption that the ankle anatomy is subverted in a joint effected by osteoarthrosis [1].

In fact, in an arthritic ankle, the talus is flattened, with a reduction in the height of the radius of curvature of 2–3 mm on average. On the tibial side, instead, the tibial plafond becomes 3 mm wider in anteroposterior orientation [10]. The Exactech Vantage^®^ is characterized by a talar resurfacing component, such as to correct the loss in length of the radius of the talar curve. On the one hand, the tibial component is significantly larger than the comparable counterparts available and this allows it to have a transmission of load forces purely on the tibial cortical, and to reduce irritation of the soft tissues. In addition, the structure of the tibial component is made so that the possible risk of damage to the anterior tibial cortical is reduced, as well as the risk of osteolysis and the formation of subchondral cysts caused by high stress-shielding (such as in Salto-Talaris and STAR). The tibial component is also designed to maximize stability and osteointegration on the tibia, using a larger central peg and three smaller anti-rotational pins to ensure long-term stability. A mobile-bearing meniscus, made with high-resistance polyethylene, is positioned between the talar and the tibial component.

The mobile meniscus allows to achieve better biomechanics and a reduction in peak pressures, but it requires accessory surgical time for balancing. There are recent papers in the literature comparing, even on a high level of evidence, fixed and movable meniscus prostheses. However, these studies have shown that patient-reported and clinical outcomes are successful for both designs and there is no significant difference in clinical improvement between the two implants [11]. It is a matter of approach and philosophy. The authors prefer to use the mobile meniscus, especially in young, sporty patients. Anyway, the fixed-bearing components were not available in Italy at the time this article was written. 

The possible complications that may occur during surgery are related to the positioning of the implant and to the associated procedures. The former includes malleolar fractures, axial deviations of the prosthetic implant, wound and periprosthetic infection, wound healing delay, and aseptic loosening. The latter includes Achilles tendon rupture, fixation devices failure, arthrodesis or osteotomy non-union.

In our series, five (12.8%) cases of delayed wound healing were reported. No cases of septic or aseptic loosening were recorded. 

A significant finding in this work is that clinical outcomes, such as validate AOFAS [12] ankle-hindfoot scores and VAS for pain measure and joint motility, were analyzed for each subject, and follow-up radiological data were available for all the patients. The improvement in the clinical condition, described by AOFAS and VAS scores, and the improvement in joint motility, as measured in dorsiflexion and plantarflexion at a one year follow-up, have shown high values in clinical tests and post-operative mobility, better or comparable to other case series in the literature and compared to the lateral surgical way for the ankle replacement. As reported by Valderrabano et al., short-term follow-up outcomes have highlighted an improvement in the AOFAS score from a mean value of 42.12 to 96.02, confirming our clinical findings. Regarding a totally different TAR approach, Mosca et al. (2021) and Usuelli et al. (2019) showed how the AOFAS score improved from an average preoperative mean value of 33.8 to a post-operative mean value of 88.5 [13,14,15]. Our findings and our clinical results appear comparable, even superior, compared to other similar and recent studies in the literature.

In our study, 14 (35.8%) of the patients underwent an associated procedure. Currently, additional procedures are performed with the implantation of the prosthesis in a single-stage surgery. Although this results in longer recovery times, it is essential for the proper alignment and the balancing of the implant, as well as for the durability of the implant itself. In fact, the restoration of proper biomechanics, not only of the ankle itself, but of the entire foot, allows for less wear and tear on the prosthesis and reduces the likelihood that the prosthesis will be painful [16].

The first limitation to this work is that it represents a monocentric database and all the procedures were executed by the same highly experienced surgeon. Then, the post-treatment FUP was short and the number of patients included was small. At last, this is a retrospective observational study. Further studies with larger numbers of patients, with a control group, are required to verify the efficacy of the Vantage prostheses compared to others.

## 5. Conclusions

Total ankle replacement has emerged as a strong treatment alternative to the ankle fusion in the management of primary and secondary end-stage AO. Nowadays, indications for joint replacement also include younger and active patients with severe ankle deformities.

In our work, we analyzed 39 primary ankle replacements through the anterior approach at a one-year minimum follow-up. The first results have shown improvements in clinical scores and post-surgical motility, better or comparable to other studies in the literature and to the lateral approach TAR.

The complications found in this study are in line with most of the published reports by other authors, with the goal of reducing them in the future. All the findings registered suggest that total ankle replacement is a safe and effective treatment option at a mean short-term observation. In any case, this study reports preliminary results of a new prosthetic design, and for this, further studies with greater follow-up and wider cohorts of patients will be necessary to confirm the reported data.

## Data Availability

Public data availability is not allowed. Data was recovered from institutional medical files.
